# STIGMA: Single-cell tissue-specific gene prioritization using machine learning

**DOI:** 10.1016/j.ajhg.2023.12.011

**Published:** 2024-01-15

**Authors:** Saranya Balachandran, Cesar A. Prada-Medina, Martin A. Mensah, Juliane Glaser, Naseebullah Kakar, Inga Nagel, Jelena Pozojevic, Enrique Audain, Marc-Phillip Hitz, Martin Kircher, Varun K.A. Sreenivasan, Malte Spielmann

**Affiliations:** 1 Institute of Human Genetics, University Hospital Schleswig-Holstein, University of Lübeck and Kiel University, Lübeck, Germany; 2 Human Molecular Genetics Group, Max Planck Institute for Molecular Genetics, 14195 Berlin, Germany; 3 Institut für Medizinische Genetik und Humangenetik, Charité - Universitätsmedizin Berlin, corporate member of Freie Universität Berlin and Humboldt-Universität zu Berlin, Augustenburger Platz 1, 13353 Berlin, Germany; 4 BIH Charité Digital Clinician Scientist Program, BIH Biomedical Innovation Academy, Anna-Louisa-Karsch-Strasse 2, 10178 Berlin, Germany; 5 RG Development & Disease, Max Planck Institute for Molecular Genetics, 14195 Berlin, Germany; 6 Department of Biotechnology, BUITEMS, Quetta, Pakistan; 7 Institute of Medical Genetics, Carl von Ossietzky University, 26129 Oldenburg, Germany; 8 DZHK e.V. (German Center for Cardiovascular Research), Partner Site Hamburg/Kiel/Lübeck; 9 Department of Congenital Heart Disease and Pediatric Cardiology, University Hospital of Schleswig-Holstein, 24105 Kiel, Germany; 10 Development and Disease, Max Planck Institute for Molecular Genetics, 14195 Berlin, Germany

**Keywords:** gene prioritzation, single-cell sequencing, congenital limb malformations, congenital heart disease, pseudotime, gene expression, congenital diseases

## Abstract

Clinical exome and genome sequencing have revolutionized the understanding of human disease genetics. Yet many genes remain functionally uncharacterized, complicating the establishment of causal disease links for genetic variants. While several scoring methods have been devised to prioritize these candidate genes, these methods fall short of capturing the expression heterogeneity across cell subpopulations within tissues. Here, we introduce single-cell tissue-specific gene prioritization using machine learning (STIGMA), an approach that leverages single-cell RNA-seq (scRNA-seq) data to prioritize candidate genes associated with rare congenital diseases. STIGMA prioritizes genes by learning the temporal dynamics of gene expression across cell types during healthy organogenesis. To assess the efficacy of our framework, we applied STIGMA to mouse limb and human fetal heart scRNA-seq datasets. In a cohort of individuals with congenital limb malformation, STIGMA prioritized 469 variants in 345 genes, with *UBA2* as a notable example. For congenital heart defects, we detected 34 genes harboring nonsynonymous *de novo* variants (nsDNVs) in two or more individuals from a set of 7,958 individuals, including the ortholog of *Prdm1*, which is associated with hypoplastic left ventricle and hypoplastic aortic arch. Overall, our findings demonstrate that STIGMA effectively prioritizes tissue-specific candidate genes by utilizing single-cell transcriptome data. The ability to capture the heterogeneity of gene expression across cell populations makes STIGMA a powerful tool for the discovery of disease-associated genes and facilitates the identification of causal variants underlying human genetic disorders.

## Graphical abstract

**Figure:**
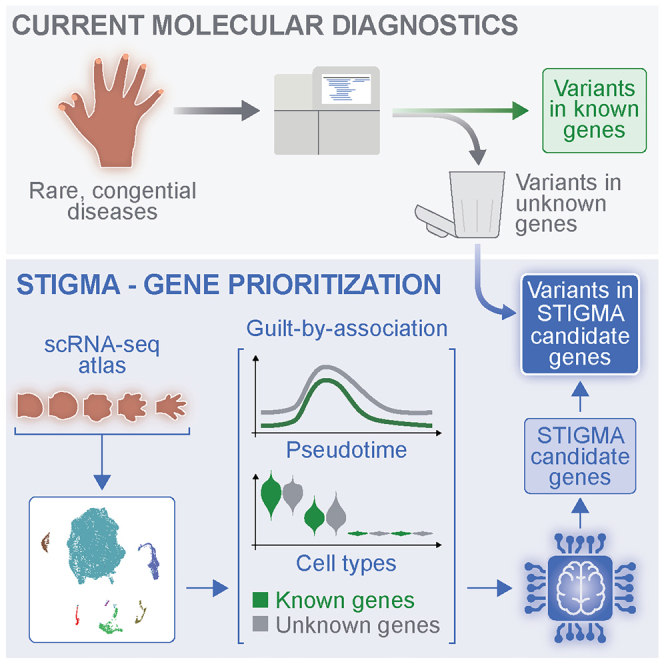

## Teaser

Single-cell tissue-specific gene prioritization using machine learning (STIGMA) is an approach to prioritize candidate genes for congenital diseases. STIGMA uses single-cell RNA-seq data to capture the dynamics of gene expression within cell populations across developmental time, making it a powerful tool for the discovery of disease-associated genes.

## Introduction

The widespread introduction of next-generation sequencing approaches has rendered the analysis of genes a routine in the clinical setting. It has benefited the ongoing discovery, functional annotation, and disease mappings of genes (e.g., HPO,^1^ OMIM^2^)^3^ as well as improvements in tools and resources to call, annotate, prioritize, and filter variants within these genes (e.g., gnomAD,^4^ DECIPHER^5^). As a result, the diagnostic yield with genome or exome sequencing has been steadily increasing, recently reaching 41%.^3^ However, to date, a causal disease link has been established for variants in only about 5,000 genes.^2^^,^^6^ Consequently, many potentially deleterious variants in genes of unknown function are classified as variants of uncertain significance (VUSs) and do not contribute to a diagnosis of rare diseases, until further validated by experimental verification, e.g., using *in situ* hybridization. In other words, incomplete gene-disease associations remain a significant bottleneck in finding a molecular diagnosis in individuals with rare genetic diseases. Gene prioritization can help overcome this limitation.^7^^,^^8^

Gene prioritization refers to arranging genes in the order of probability of association with a disease. It can help narrow down the list of candidate genes under consideration. Gene prioritization usually requires prior knowledge about the genes, including (1) a list of seed genes that are known to be associated with the disease and (2) data on the genes/proteins, such as protein-protein interactions, gene expression profiles, known functional annotations (ontology, pathways etc.), disease-gene associations,^9^ and intrinsic gene properties (genomic position, sequence, GC content, conservation, structure, etc.).^10^ A computational model then assigns a “disease-causing” probability to every gene either based on existing annotations for that gene or based on “guilt by association” with known disease-associated genes in interacting networks or machine learning models.^7^ The tools that rely on functional annotations or disease associations of the gene being prioritized are often heavily biased toward highly characterized genes.^8^ Such methods have also been reported to yield false positive predictions due to evolving disease-gene associations.^11^ In contrast, tools such as GeneFriends,^12^ GADO,^13^ EvoTol,^14^ and GeneTIER^15^ that rely exclusively on gene expression data, evolutionary intolerance, and intrinsic gene data are inherently unbiased. For example, GeneTIER is based on the hypothesis that “genes responsible for a tissue-specific phenotype are expected to be more highly expressed in affected than unaffected tissues.”^15^^,^^16^ Such annotation-agnostic tools can prioritize candidate genes lacking functional annotations.^10^ However, most current gene expression-based prioritization tools use bulk-RNA sequencing (bulkRNA-seq) data (e.g., GTEx^17^) containing expression profiles at an organ-level resolution.^18^ This introduces two major issues with regards to the specificity of gene expression. Firstly, the expression of cell type-specific genes are averaged out in these datasets. Secondly, such approaches do not explicitly consider the temporal dynamics of expression, which is crucial during organogenesis. In the context of diagnosing a rare congenital disease, this can lead to the current approaches being non-specific and insensitive. For instance, the inability to predict a known disease-gene association in the case of Parkinsonism-dystonia (MIM: 613135) was attributed by the authors to the highly cell type-specific expression of *SLC6A3* (MIM: 126455).^13^ Using cell type- and developmental time-specific gene-expression data could improve the gene prioritization outcome.

The boom of single-cell sequencing (sc-seq) has enabled the creation of cell atlases of humans and model organisms, providing reference maps with cell types, cell states, their gene expression profiles, spatial location, and chromatin profiles throughout embryogenesis and adulthood.^19^^,^^20^^,^^21^^,^^22^ The technology has enabled a more in-depth analysis of molecular mechanisms throughout a lifetime (i.e., from embryogenesis through birth to old age) in states of health and disease at cellular resolution and is transforming healthcare.^23^^,^^24^^,^^25^^,^^26^^,^^27^^,^^28^ These cell atlases are already being used to prioritize variants or to establish variant-to-function mappings.^29^ However, to the best of our knowledge, single-cell RNA sequencing (scRNA-seq) data have not yet been applied for gene prioritization, where the cell type-specific or developmental stage-specific expression profiles are taken into consideration. Arguably, the only exception is a recently published risk gene identification method, VBASS.^30^ VBASS uses scRNA-seq data to identify disease-associated genes from *de novo* variant data from large cohorts. In contrast, the goal of gene prioritization as discussed here is to narrow down the list of candidate genes in rare disorders or a single individual.

Here, we introduce scRNA-seq data-based gene prioritization for congenital diseases by developing single-cell tissue-specific gene prioritization using machine learning (STIGMA). STIGMA predicts the disease-causing probability of genes based on their expression profiles across cell types, while considering the temporal dynamics during the embryogenesis of a healthy (wild-type) organism, as well as several intrinsic gene properties. We validate our approach by applying the model on mouse limb and human fetal heart scRNA-seq datasets, to prioritize genes for congenital limb malformations and congenital heart disease (CHD), respectively. STIGMA successfully predicted several gene-disease associations, such as *UBA2* (MIM: 613295), which was recently reported to be related to limb malformations,^31^ as well as *ALDOB* (MIM: 612724) and *MMP9* (MIM: 120361) that have been associated with ventricular septal defect (MIM: 614429).^32^^,^^33^ It also suggested *PRDM1* (MIM: 603423), the ortholog of which has been shown to be associated with hypoplastic left ventricle and hypoplastic aortic arch in mouse models (MGI: J:175213).

## Material and methods

### Preparation of mouse limb scRNA-seq data

All of the following steps were carried out using cellranger (v.3.0, 10× Genomics),^34^ scrublet (v.3),^35^ seurat (v.3),^36^ biomaRt (v.2.46.3),^37^ splines (v.4.0.0),^38^ and monocle3^39^ as well as standard packages for R (v.4.0.5) and python (v.3.7.4).

The wild-type mouse scRNA-seq data are a combination of a dataset generated in this study and published datasets. The generated data originate from forelimbs (E9.5 to E12.5) and hindlimbs (E11.5 to E12.5) and it was combined with published scRNA-seq datasets of the forelimb between time points E10.5 and E15.0 from ENCODE accession ENCSR713GIS^40^ (fastq files) and of the hindlimb between time points E11.5 and E18.5 from GEO accession GEO: GSE142425^41^ (gene-barcode UMI count matrices).

When the UMI count matrix was not available, cellranger^34^ was used with default parameters to generate it from the fastq files. Scrublet^35^ was used to detect doublets and only cells with doublet scores below 0.2 were retained for the analysis. Further, only cells with more than 1,000 UMI and 500 genes and less than 10% mitochondrial DNA and 50% of ribosomal gene content were retained. Ribosomal and mitochondrial genes were removed for calculating cell embeddings. The data were normalized using *SCTransform* function in seurat^36^ with 6,000 highly variable genes (hvgs). At this point, the datasets from the three sources were integrated to remove batch effects using the built-in integration pipeline in seurat^35^^,^^36^^,^^42^ based on 1,000 genes as integration anchors. Principal component (PC) analysis based on the top 1,000 hvgs was performed on the integrated data to reduce the dimensionality. The nearest neighbors cell-cell graph built using the top 50 PCs was clustered using the Louvain algorithm,^43^ with a resolution of 0.05. Cell-type marker genes were identified by differential expression (DE) analysis using the ROC approach implemented in the *FindAllMarker* function in seurat. DE analysis was performed on genes passing the cut offs of average fold change (|avg_logFC| > 0.25) and percentage of cells expressing the gene per cluster (min.pct > 0.1). The DE genes were used to annotate the main clusters. The clusters (immune cells, neuronal cells, vascular cells, and erythrocytes) that represented less than 4% of the data and those that were deemed not to generate limb-specific congenital malformations were removed. The remaining clusters were further sub-clustered (muscle cells: nhvg = 500, npcs = 20; ectoderm: nhvg = 500, npcs = 20; mesenchyme: nhvg = 1,000, npcs = 35) and annotated as before. Several characteristics of gene expression were also calculated using seurat to be used as STIGMA-classification features for each gene. These included mean expression in each sub-cluster (*AverageExpression*), variance in expression within each sub-cluster (*HVFInfo*), the percentage cells expressing the gene in each sub-cluster (*PrctCellExpringGene*), and the fold-change in expression between each sub-cluster and the rest of the cells (*FoldChange*). Only genes that had an average expression greater than 0 in at least 1 of the cell types were retained.

Trajectory analysis to capture the gene expression dynamics was performed separately for each sub-cluster using the monocle3^39^ workflow. The cells were ordered using *order_cells* with the earliest embryonic time point set as the root. The resulting pseudo time data were pooled into 20 bins and the average expression of the genes in each of these bins was calculated. To adapt this temporal data into a feature for random forest classification, it was fitted to a cubic spline function with 10 control points using the *bs* function of the splines package. The coefficients of the spline were obtained for each of the genes per cluster by solving the least squares fit and used as input features for the model.

### Preparation of human fetal heart scRNA-seq data

Analyses were carried out using Seurat (v.4), splines (v.4.0.0), monocle3^39^ as well as standard packages for R (v.4.0.5) and python (v.3.7.4). The human cell atlas of fetal gene expression consisted of 101,748 cells from 121 human fetal samples with data from the heart, ranging from 90 to 122 days post-conception.^19^ Data were downloaded as a loom file and contained 16 annotated cell types. Only the cell types representing at least 1.5% of the data were retained. The remaining processing of the dataset, like calculating the gene features per cluster, was identical to that of mouse limb scRNA-seq data described above.

### Intrinsic gene properties as features for classification

Processing steps were carried out using the R packages biomaRt (v.2.46.3), GenomicFeatures (v.3.10.0),^44^ BSgenome.Hsapiens.UCSC.hg38 (v.1.4.3),^45^ and Repitools v.1.36.0^46^ for R (v.4.0.5). Gene constraints such as pLI, pNull, pRec, syn_Z, mis_Z, and lof_Z metrics for protein-coding genes were downloaded from gnomAD (v.2.1.1).^4^ When absent and for non-coding genes, these metrics were imputed (see steps 1–3 in classification pipeline). To estimate the GC content of each gene and its upstream promoter region, the list of known genes for the human genome build hg38/GRCh38 was obtained using the BSgenome.Hsapiens.UCSC.hg38 library. For every gene, the promoter sequence, spanning 500 base pairs upstream and 100 base pairs downstream of the transcription start site, was obtained using the *promoters* function on the BSgenome.Hsapiens.UCSC.hg38 object. The percentage GC content in the gene and the promoter sequences were separately estimated using *gcContentCalc* and used as classifier features for the genes. Additionally for the limb dataset, mouse human ortholog confidence (BioMart) was included as input feature.^37^^,^^47^

### Positive and negative classes for congenital limb malformation

The green list of genes associated with “Limb Disorders” (PanelApp v.2.0, downloaded on 23 June 2021)^48^ was filtered to include only genes that show cell type specificity in expression. The average expression of the genes was quantified for each sub-trajectory within epithelial, hepatic, and mesenchyme trajectories in the mouse organogenesis cell atlas.^23^ If a gene had the same expression (SD ± 1) in more than 10 sub-trajectories, they were filtered out. The negative training set was composed of housekeeping genes that were LoF tolerant based on gnomAD (pNull > pRec and pNull > pLI).^49^^,^^50^

### Positive and negative classes for congenital heart disease

A curated list of genes known to be associated with congenital heart disease,^51^ whose average expression was not ubiquitous across epithelial, hepatic, mesenchyme trajectories in the mouse organogenesis cell atlas,^23^ was used for the positive class of the training set. As before for the predictions on the limb dataset, housekeeping genes that were LoF tolerant based on gnomAD (pNull > pRec and pNull > pLI) were used as the negative training set.^49^^,^^50^

### Classification pipeline

The following steps were carried out using the sklearn (v.0.24.2)^52^ package for python (v.3.7.4). A pipeline was set up using the *make_pipeline* function to optimize the parameters of the classifier. The classification workflow consisted of the following steps: (1) iterative imputing, (2) scaling, (3) synthetic oversampling, and (4) generating the random forest model. Missing data in the dataset were imputed using the *IterativeImputer* from scikit-learn with default parameters. The data were scaled using the *MinMaxScaler*. The class imbalance in the positive and the negative classes was corrected by synthetic minority over-sampling using an adaptive synthetic (SMOTE-ADASYN) algorithm.^53^ This algorithm was chosen because it creates a synthetic representative dataset rather than simply duplicating the minor dataset. The best parameters for synthetic oversampling (n_neighbors) and the random forest model (n_estimators, max_depth, min_samples_split, min_samples_leaf) were optimized using *GridSearchCV* based on recall (for congenital limb malformations: adasyn: n_neighbor = 10, randomforest: n_estimators = 130, max_depth = 15, min_samples_split = 2, min_samples_leaf = 1 and for congenital heart disease: adasyn: n_neighbor = 5, randomforest: n_estimators = 90, max_depth = 30, min_samples_split = 5, min_samples_leaf = 1).

The final random forest model was built based on these optimized parameters and bootstrap resampling. Features that were significant for the performance of the model were obtained using the attribute *feature_importances_*. 5-fold cross-validation was used to calculate the out-of-bag error to validate the model and to avoid overfitting. The trained model was used to classify all genes. Those represented in the training classes were later removed from the predicted list. The area under the curve and other ROC metrics were calculated using the *roc_curve* function of sklearn.metrics. The threshold was chosen by plotting the density graph of the validation dataset (Figures 2F and 3D). The probability at which the negative class was at 0 density was chosen as the threshold. It is worth noting that the duplicated use of the same dataset for parameter optimization and validation likely leads to slightly inflated ROC metrics. The relatively small number of high-confidence positive class genes made the creation of a dedicated hold-out set for model validation impractical. However, this limitation can be overcome in the future as more genes acquire phenotypic annotations.

### UMAP embedding of training classes based on input features

The input data were imputed, scaled, and class balanced as stated before. The UMAP object was constructed using the UMAP library of python. The *fit_transform* method of the UMAP class learns the embedding and transforms it to a numpy array, which is then plotted using the scatterplot method of plotly.

### Explorative analysis based on Monarch Initiative

All gene phenotypes were downloaded from the Monarch Initiative Explorer.^54^ Disease-specific ontology terms were downloaded from MouseMine.^55^ Fisher’s exact test was performed to verify the significance of the association between phenotype and STIGMA ranking.

## Results

### STIGMA model setup

**Figure 1:**
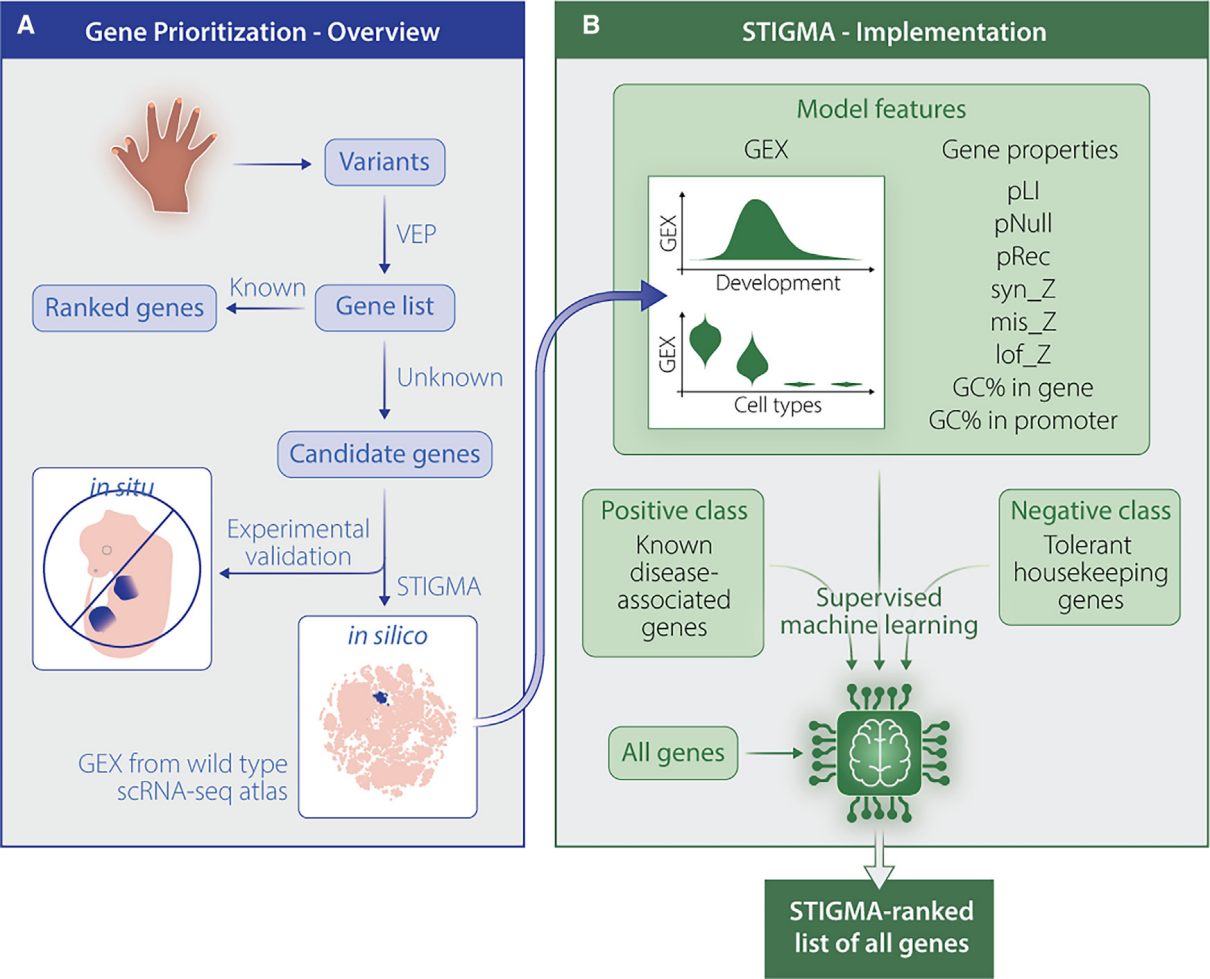
Implementation of gene prioritization within STIGMA for congenital diseases (A) The genetic diagnostic workflow for congenital diseases (e.g., limb malformations) comprises the detection of variants and their prioritization, often resulting in many candidate genes that necessitate experimental validation. STIGMA enables the prioritization of the candidate genes with the use of development cell atlases of wild-type model organisms. (B) In STIGMA, supervised machine learning is applied to the single-cell gene expression data as well as intrinsic gene properties (e.g., pLI, lof_z) on positive and negative classes. The probability of pathogenicity is then predicted for all genes (including genes lacking functional annotations) resulting in a ranked list of genes. GEX represents gene expression.

Since we set out to predict the probability of every gene to be associated with the disease of interest, including those with little or no prior functional annotation, STIGMA was designed to use only scRNA-seq data and gene-intrinsic properties as features for model training and prediction (Figure 1A). The scRNA-seq data from wild-type samples during embryonic development were obtained from published datasets as well as datasets generated in this study. Gene expression in these datasets was encoded at the cell cluster-level to represent cell type specificity and developmental dynamics. Gene-level metrics per cluster included mean, variance, fold change compared to the rest of the cells, and the fraction of expressing cells. Developmental dynamics were captured by organizing the cells along a pseudo-temporal developmental trajectory and aggregating the gene expression along pseudo-time bins.

The gene-intrinsic properties included gene constraint metrics from gnomAD and GC content of the gene as well as its promoter. The gene constraint metrics used were related to the genes’ (in)tolerance to LoF, synonymous, or missense variants, specifically the pLI (probability of being intolerant to LoF heterozygous variants), pRec (probability of being intolerant to LoF homozygous variants), pNull (probability of being tolerant to LoF variants), syn_Z (*Z* score of the number of synonymous variants in gene), mis_Z (*Z* score of the number of missense variants in gene), and lof_Z (*Z* score of the number of LoF variants in gene)^49^ scores.

The supervised learning of these features in STIGMA was implemented using a random forest classifier^52^^,^^56^ (Figure 1B). This machine learning algorithm has been widely used in disease classification due to its ensemble property that allows combining predictions from multiple decision trees and due to its interpretability.^57^^,^^58^ This choice was also based on our preliminary tests on other algorithms, such as support vector machines, which yielded suboptimal validation outcomes (e.g., precision = 0.413). The model was trained on the aforementioned features using two classes of genes: (1) a positive class, composed of genes known to be associated with the disease of interest and (2) a negative class, composed of housekeeping^50^ genes that were more probable to be “tolerable” to LoF than being intolerant to homozygous or heterozygous LoF (i.e., pNull > pRec and pNull > pLI).^49^ Due to the ubiquitous expression of housekeeping genes, STIGMA will likely not prioritize genes with syndromic phenotypes. Conversely, congenital diseases, which are the focus of STIGMA, are most likely caused by the LoF of genes crucial to the development of a distinctive organ and will likely exhibit increased temporal and/or tissue-level expression specificity.^59^ The model performance in terms of accuracy, sensitivity, and precision was evaluated using a 5-fold cross-validation approach. Separate models were generated to prioritize genes for each of the two congenital disease groups, with disease-specific positive class and the associated model features.

### STIGMA for congenital limb malformations

**Figure 2:**
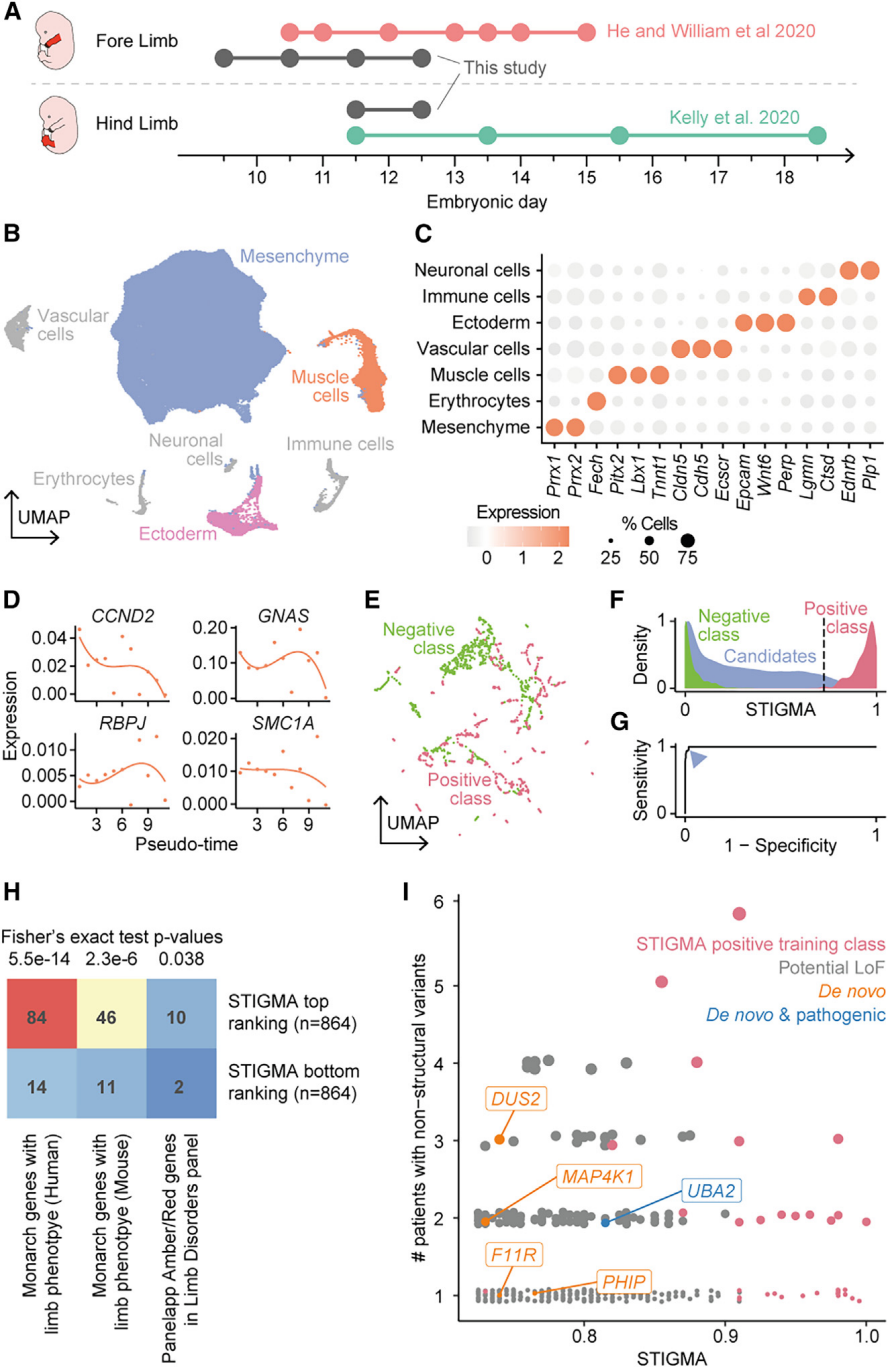
scRNA-seq dataset and performance of the disease-gene classifier for congenital limb malformations (A) Embryonic time points represented across the scRNA-seq datasets.^40^^,^^41^ (B) 2D UMAP embedding of the major cell types after batch correction across the datasets, where points represent cells. Cell types not used for training STIGMA are grayed out. (C) Marker genes corresponding to the cell types in (B). (D) Dynamics in the gene expression of a representative set of positive class genes in muscle cells along the developmental pseudo-time. Points represent the spline knots and lines represent spline fits. (E) 2D UMAP embedding of the genes in the training dataset, including those imputed for class balancing, based on the input features used for STIGMA. (F) Distribution of STIGMA scores for training classes and candidate genes. Dotted line marks the threshold of 0.725. (G) ROC curve (AUC 0.99) showing the performance of the model. The arrowhead marks the threshold of 0.725. (H) Number of genes ranked top or bottom in STIGMA (excluding the training class), with at least one associated limb phenotype in Monarch or those being members of the Limb Disorder panel of PanelApp (classified Amber or Red). p values of Fisher’s exact test are provided. The 95% confidence interval of the odds ratio did not cross unity for the three tests. (I) STIGMA scores of genes predicted to be disease associated and featuring potential LoF variants in a previously published cohort of 69 individuals with limb malformations.^31^ Genes containing *de novo* variants, including those identified to be pathogenic in the study, are highlighted.

First, we trained STIGMA to predict genes associated with congenital limb malformations. Congenital limb malformations were chosen since the diagnostic yield is currently quite low, at less than 20%, and candidate genes are likely to have a distinct cell type-specific expression in the limb. scRNA-seq data were compiled from three mouse limb datasets, two published^40^^,^^41^ and one from this study across embryonic days E9.5 to E18.5, spanning the period of limb development from the appearance of limb buds to interdigital separation and the completion of the limb outgrowth.^60^ The data represented a total of 151,444 cells and 40,098 genes of which 19,571 had a human ortholog. Standard analysis including dimensionality reduction, clustering, and differential gene expression analysis revealed seven main cell types (Figures 2A and 2B), which were annotated based on marker genes (Figure 2C). Next, we reduced the dataset to contain only mesenchyme, ectoderm, and muscle cells by removing immune cells, neuronal cells, vascular cells, and erythrocytes, which have not been described to cause limb-specific congenital morphological malformations.^61^ The final dataset contained 144,266 cells. Further sub-clustering to increase the cell type specificity of gene expression profiles led to two ectoderm sub-clusters and four mesenchyme sub-clusters, which were manually annotated (Figure S1). Pseudo-bulk gene expression of every gene was calculated per sub-cluster at several pseudo-time bins (Figure 2D).

The positive class of genes (n = 88) was a subset of the diagnostic-grade “green” list of genes in the panel “Limb Disorders” from the Genomics England PanelApp.^48^ We removed genes that showed pervasive expression in all trajectories in the mouse organogenesis cell atlas (MOCA) (Figure S2),^23^ resulting in 87 genes in the positive class. Tolerant housekeeping genes (643 genes) were used for the negative class (Table S1 containing gene lists of both classes). Class imbalance-correction by SMOTE resulted in a size of 643 for both classes. To verify whether positive and negative classes segregate based on the selected model features, we visualized the genes by projecting all the input features onto a 2D uniform manifold approximation and projection (UMAP), which showed a clear segregation between the two classes (Figure 2E), suggesting that a classification based on the features included in the model was appropriate.

Next, we used the positive and negative training classes to optimize the hyperparameters of the classifier using GridSearchCV. The hyperparameter-optimized model was trained using 5-fold cross validation. The receiver operator characteristic (ROC) curve, where the sensitivity (true positive rate) is plotted against 1-specificity (false positive rate), had an area under the curve (AUC) of 0.99 (Figures 2F and 2G). At a threshold STIGMA score (disease-causing probability) of 0.725, the sensitivity and the precision of the binary classifier reached 0.9545 and 0.875, respectively. Application of the final model trained on single-cell features on all genes resulted in 864 STIGMA-predicted candidate genes (SCGs) associated with congenital limb malformations with STIGMA scores greater than 0.725 (Table S2).

Since the random forest model lends itself to the analysis of relative importance of the various features that contribute to the classifier, we wondered to what extent the single-cell features influenced the model. Including pseudotime features, the single-cell features had a feature importance mean square of 3.25 to contrast with a value of 0.01 for gene-intrinsic properties (Figure S3A). In other words, the STIGMA score that each gene receives is based on the cell type-specific temporal dynamics in gene expression and, to a smaller extent, is based on the gene-intrinsic metrics, including the population-level constraint metrics. We also confirmed the importance of single-cell data for the performance of STIGMA by training on pseudo-bulkRNA-seq data generated from the same dataset. This resulted in a dramatic drop in performance, leading to the misclassification of nearly 40% of the positive class genes. Moreover, as can be expected from the feature importance plot, the cell type-specific expression alone is also insufficient to classify the genes (Figure S4). Together, these analyses indicated the combined importance of cell type-specific and pseudotime-specific gene expression information.

We verified these SCGs by several means. Firstly, we systematically explored the phenotypes reported for the SCGs and non-SCGs by the Monarch Initiative,^54^ a portal for genotype-phenotype data across multiple species, with the rationale to expect enrichment of genes with limb-associated phenotypes in the top-ranking STIGMA genes. Indeed, this analysis (Figure 2H; Table S3) showed a significant enrichment of genes with at least one limb-associated phenotype in SCGs when compared to bottom ranking genes in both human (84 vs. 14 genes) and mouse (46 vs. 11), with Fisher’s exact test p values of 5.5e−14 and 2.3e−6, respectively. Secondly, we checked the representation of genes labeled “Amber” (borderline evidence) and “Red” (low level of evidence) in the Limb Disorders panel of PanelApp in the STIGMA top and bottom ranking genes. This also showed a 5-fold enrichment (10 vs. 2), with a Fisher’s exact test p value of 0.038.

As a final means of validation, we performed a manual search through the literature for reports where the SCGs were associated with limb disorders. This led to the identification of 112 SCGs, which were either genes with known association with congenital limb malformations, but not yet in the PanelApp green list (and by extension not in our positive training class), or genes that had nominal evidence in the literature (Table S3). For example, genes that were assigned a disease probability of greater than 0.9 included *HAS2* (MIM: 601636) and *FGFR3* (MIM: 134934), which are known to be associated with limb malformations.^62^^,^^63^ While *FGFR3* is on the PanelApp green list (limb disorders), it was not included in STIGMA’s positive class training list, because of its ubiquitous expression in MOCA. Another example is *UBA2*, which was ranked 309 by STIGMA with a probability of 0.81, and was recently reported to be associated with ectrodactyly (MIM: 619959).^31^^,^^64^ In addition, as a means of further validation, we also identified several genes that carried potential LoF mutations in a cohort study of undiagnosed individuals with congenital limb malformations (Figure 2I).^31^ Of the 7,082 potential rare non-structural LoF variants identified by genome sequencing in 69 individuals with congenital limb defects,^31^ 469 variants were found in 345 genes with STIGMA scores higher than the classification threshold of 0.725. These comprised eight of the nine genes found to carry likely pathogenic variants in the original study, including well-described genes with variants previously associated with limb disorders, such as *HOXD13* (MIM: 142989) and *GLI3* (MIM: 165240) from the positive training class as well as the STIGMA-predicted CG *UBA2*, described above, and missing only *HMGB1* (probably because of its ubiquitous expression, Figure S2). Notably, five genes implicated by STIGMA featured *de novo* variants in this dataset, which were not identified as potentially pathogenic in the original study (*DUS2* [MIM: 609707], *MAP4K1* [MIM: 601983], *F11R* [MIM: 605721], *PHIP* [MIM: 612870], and *LRP4* [MIM: 604270]). Only two of these genes have been previously associated with diseases: *LRP4* and *PHIP*. *LRP4* is associated with autosomal-recessive Cenani-Lenz syndactyly syndrome (CLS [MIM: 212780]) and was in our positive training class. *PHIP* was not part of the positive training class (absent in PanelApp) and is associated with autosomal-dominant Chung-Jansen syndrome (MIM: 617991), a phenotype comprising intellectual disability, obesity, dysmorphic facial features, notably tapering fingers, and clino- and syndactyly. The *PHIP* variant occurred in an individual with a complex malformation syndrome including renal agenesis, hypoplastic radii, oligodactyly of the hands, and polydactyly of the feet. Interestingly, *PHIP* and *UBA2*, which have been associated with similar disease profiles of oligodactyly and ectrodactyly, respectively, also showed similar temporal expression patterns in mesenchymal-chondrocytes, -fibroblasts, ectodermal-sost, and muscle cells (Figures S5B, S5D, S5E, and S5G). *DUS2*, *MAP4K1*, and *F11R,* which were not previously associated with any inheritable disease, were identified by STIGMA to be promising candidate genes from this cohort. *DUS2* variant was found in some individuals who also carried the LRP4 variant. Variants in *MAP4K1* and *F11R* were found in an individual with syndactyly of the hands and feet and in an individual with forearm reduction defects, respectively. Whether these genes are additionally associated with these phenotypes remains to be determined.

### STIGMA for congenital heart diseases

**Figure 3:**
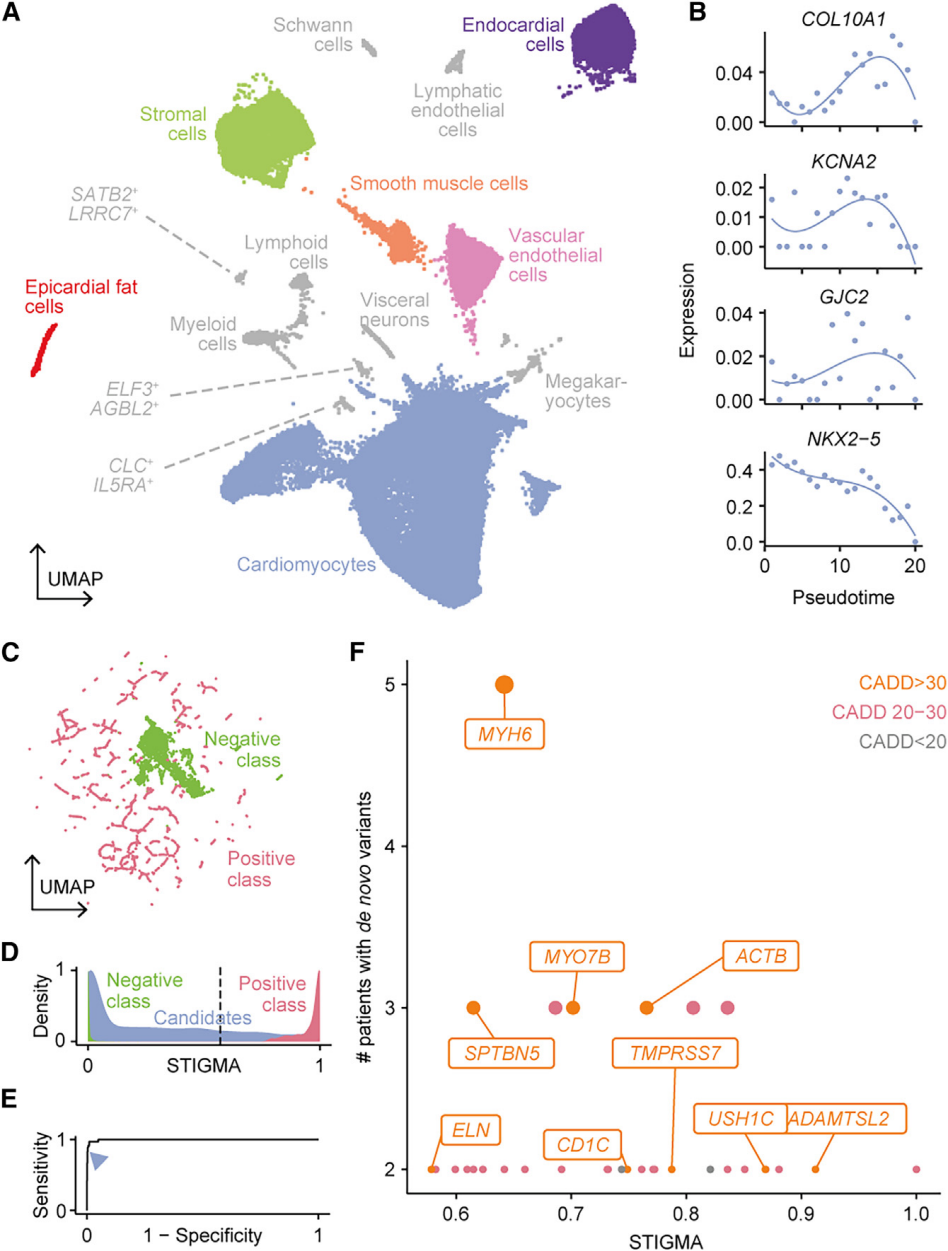
scRNA-seq dataset and performance of the disease-gene classifier for congenital heart disease (A) 2D UMAP embedding of the cells, where the colors indicate the cell type annotations. Cell types not used for training STIGMA are grayed out. (B) Dynamics in the gene expression of representative positive class genes along the developmental pseudo-time. Points represent the spline knots within a sub-cluster and lines represent cubic spline fits. (C) 2D UMAP embedding of the genes in the training dataset, including those imputed for class balancing, based on the input features used for the STIGMA. (D) Distribution of STIGMA scores for training classes and candidate genes. Dotted line marks the threshold of 0.57. (E) ROC curve (AUC 0.9972) showing the performance of the model. The arrowhead marks the threshold of 0.57. (F) STIGMA scores of genes featuring disruptive *de novo* variants in a previously published cohort of 2,489 trios with congenital heart disease.^51^ Only SCGs with at least two *de novo* variants are plotted.

Given the performance of STIGMA for congenital limb malformations, we extended the approach to predict genes associated with congenital heart diseases (CHDs).^51^ After downloading and filtering, the scRNA-seq dataset^19^ contained expression values of 63,561 genes in 101,749 cells, within 16 annotated cell types, of which the cardiomyocytes represented the largest cluster, containing 66% of cells in the dataset (Figure 3A). Removal of cell types such as lymphoid cells and visceral neurons, which have not been reported to lead to congenital heart disease,^65^ resulted in 96,276 cells across 6 cell types. As before, the gene-expression values across these cell types and along the pseudo-time bins in addition to gene intrinsic features were used as input features for training STIGMA (Figure 3B).

A manually curated list of genes (n = 331) known to be associated with congenital heart disease was used as the positive class of the training set.^51^ As before for the predictions on the limb dataset, 643 tolerant housekeeping genes were used as the negative training set. When the complete list of curated disease-causing genes were used to train and run the model, STIGMA predicted 12,012 genes potentially associated with CHD, with a precision of 0.8067. To improve the precision and to reduce the number of SCGs, we analyzed the positive class genes based on their expression pattern in other tissues in MOCA.^23^ This revealed several ubiquitously expressed genes (UEGs) whose removal resulted in as few as 36 genes in the positive class (Table S1). As before, the positive and negative class genes for CHD demonstrated good separation based on the input training features, as visualized by a UMAP embedding, confirming compliance to random forest classification (Figure 3C). As in the limb, the single-cell features for the cardiac disease model were considered important for the model performance compared to the gene-intrinsic property, with a mean square value of 0.97 for single-cell features, including pseudotime features, and 0.03 for gene properties (Figure S3B).

The hyperparameters were optimized as before, resulting in a ROC curve with an AUC of 0.9972 (Figure 3E). A low number of genes in the positive training class resulted in a skewness in the distribution of the prediction probability, so a threshold of 0.57 was chosen to achieve a sensitivity above 0.8. At this chosen threshold, sensitivity and precision were 0.8333 and 0.8421, respectively (Figures 3D and 3E), predicting 3,715 SCGs to be potentially associated with CHD (Table S4).

We verified the STIGMA predictions by manually searching the literature. In an integrative study of genomic copy number variants (CNVs) and *de novo* intragenic variations (DNVs) of a CHD cohort with 4,190 DNVs (in 4,190 genes),^51^ 468 genes were among the predicted SCGs, accounting for 543 variants. Furthermore, 34 of these genes had nonsynonymous *de novo* mutations in at least two individuals, nine of which had CADD scores over 30, and 10 of which were reported to be associated with heart phenotypes (Figure 3F). For example, in humans, *ALDOB* (MIM: 612724) and *MMP9* (MIM: 120361) have been found to be associated with ventricular septal defect.^32^^,^^33^ *FLT4* (MIM: 136352) has been associated with pulmonary atresia with ventricular septal defect (MIM: 178370) at a prevalence of 0.2% and constituting 2% of the CHDs.^66^ *MYH7B* (MIM: 609928) has been associated with left ventricular non-compaction cardiomyopathy (MIM: 604169), where the muscles extending from the left ventricle to the chamber gradually transform from sponge-like to smooth and solid.^67^ Some of these genes have been implicated in heart phenotypes in mice. Namely, *Myh6* has been associated with dilated cardiomyopathy (MIM: 613252) and decreased contractile function^68^ with hypoplastic left heart syndrome (HLHS [MIM: 241550]),^69^ but is also associated with atrial septal defects (MIM: 614089) in humans.^70^ *Scn10a* has been associated with sinus bradycardia phenotype and irregular RR interval upon scruffing.^71^ *Eln* haploinsufficiency has been associated with aortic valve malformation.^72^ Finally, another SCG, *Prdm1*, is associated with hypoplastic left heart syndrome and with hypoplastic aortic arch (MGI: J:175213).

## Discussion

Exome and genome sequencing has become a valuable tool in understanding the genetic basis of human diseases, enabling the identification of genetic variants associated with various conditions.^73^ However, the sheer volume of variants detected in a single individual poses a significant challenge in distinguishing pathogenic variants from benign ones.^3^ Several approaches have been developed to aid this process, including searching through well-established databases such as 1000 Genomes, gnomAD, and ClinVar to determine the population frequencies of detected variants.^49^ Additionally, the functional impact of variants is predicted using various computational approaches, enabling the identification of potentially relevant variants.^74^

While these initial filtering steps are valuable, they primarily focus on the variant level and may yield a substantial number of candidate variants in poorly understood genes that need further evaluation or painstaking experimental validation. Computational gene prioritization methods that do not rely on prior functional/disease annotations offer an alternative to shorten the list of these candidate variants further. However, all existing gene-prioritization methods based on gene expression data use bulkRNA-seq data. Indeed, a recently published method for risk gene identification, VBASS,^30^ incorporated scRNA-seq data to improve upon previous methods^75^^,^^76^ to identify disease-associated genes in *de novo* variant data within cohorts of affected and control individuals. However, these methods are not exactly gene-prioritization methods, because they do not globally prioritize all genes. That is, unlike STIGMA, VBASS is not designed to narrow down the list of candidate genes under consideration for an individual. Overall, STIGMA addresses some of the limitations of traditional gene prioritization techniques. Specifically, STIGMA leverages recent developments of scRNA-seq to better understand the expression dynamics of genes across different cell types during organogenesis. By incorporating this information into the prioritization process, STIGMA provides a more comprehensive and tissue-specific assessment of candidate genes, making it a promising and cohort-independent tool for identifying variants in potentially disease-associated genes in an individual.

We implemented STIGMA in the context of two congenital disease groups—limb malformations and CHD. Since the genes in training classes and the features used to train the model directly influence model performance, we first verified that the features sufficiently discriminated the positive from the negative classes and then confirmed the results by cross-validation. STIGMA classified 864 and 3,678 genes to be SCGs for congenital limb malformations and heart disease, respectively.

We validated STIGMA predictions using multiple approaches. Automated analysis based on gene-phenotype data aggregated by the Monarch Initiative^54^ as well as in the PanelApp^48^ Amber/Red lists demonstrated the enrichment of genes with limb phenotypes in the top genes ranked by STIGMA. A manual search of the literature also revealed multiple lines of phenotypic evidence for the SCGs. For example, 469 LoF potential variants were found in 345 SCGs in a cohort study, with notable genes such as *UBA2*, *PHIP,* and *LRP4* not present in curated lists such as PanelApp (at the time of our download).^31^ Similarly, CNVs and *de novo* variants were present in 468 SCGs in a CHD cohort, with many such as *ALDOB*, *FLT4*, *MYH7B*, *Scn10a*, and *Eln* associated with heart phenotypes in humans or mice.^51^

Although trained merely on murine scRNA-seq data, STIGMA was able to correctly suggest genes known to cause limb malformations in humans, confirming that it is able to prioritize human genes. Indeed, a direct comparison of predictions by STIGMA models for congenital heart disease trained with comparable murine and human scRNA-seq datasets revealed a statistically significant Pearson’s correlation of 0.76 (Figure S6). Moreover, both models retrieved the same 34 genes that harbored *de novo* mutations in the cohort, confirming that a murine dataset can be a good approximation when a human dataset is unavailable. Nevertheless, the use of a future human scRNA-seq dataset is likely to improve the model predictions.^77^

Interestingly, temporal gene expression dynamics was more important in the STIGMA congenital limb malformation model than in the STIGMA CHD model. This is possibly because the murine limb scRNA-seq datasets spanning E9.5 to E18.5 match the embryonic stages most relevant to limb development (E9.5 to E14.5).^77^^,^^78^ The human heart dataset invoked in STIGMA, however, spans days 90–122 after conception,^19^ while cardiac organogenesis occurs earlier—from 26 to 56 days post conception.^79^ This could have rendered the temporal dynamics in gene expression less relevant for the CHD model. A better matched heart development dataset could improve the model outcomes to levels obtained for the limb model. Nevertheless, as implemented currently, cell type-dependent gene expression values appear to facilitate clinically relevant gene prioritization.

The approach of STIGMA, as currently implemented, also has certain limitations: the choice of genes for training affects the prediction and accuracy. STIGMA assumes that genes crucial to the development of a distinctive organ (e.g., limb) are neither ubiquitously expressed nor expressed in all cell types within that organ. However, it is possible that the assumed expressional specificity occurs only at the transcript level, which most currently available atlas-level scRNA-seq data are insensitive to.^80^^,^^81^ This could result in false negative predictions due to removal of “ubiquitously expressed genes” from the positive training class. Splice-sensitive scRNA-seq atlases that allow transcript counts rather than pooled gene counts could overcome these limitations. Additionally, the incomplete coverage of exonic LoF variants and the underrepresentation of several populations in gnomAD could have limited the functionality of STIGMA.^18^^,^^49^ Moreover, like other expression-based annotation-agnostic gene prioritization methods, STIGMA too, is based on the principle of guilt by association. This could miss genes directly associated with a disease, if their molecular mechanisms differ from those used to train the classifier. Future STIGMA versions will require updating of the positive training class as more genes are phenotypically annotated.^7^ Increased number of genes from the positive training class can also help remove biases introduced due to oversampling used to attain class balance. Contrariwise, STIGMA appears to perform reasonably well when trained with as few as ∼10 genes in the positive training class based on the performance metrics alone (Figure S7). Techniques such as VBASS, which identify risk genes based on *de novo* variants in cohorts of affected individuals, could be utilized to expand the positive training class. Here, STIGMA can also help identify risk genes that may not feature any *de novo* variants in the cohort. STIGMA, as it is currently implemented, includes intolerance metrics (from gnomAD) as model features as a means of capturing genes based on these features as well. Consequently, it is possible that the predictions are biased against potential disease-associated genes that are not under selection pressure*.* Finally, while STIGMA will benefit from a more comprehensive validation of all the predicted SCGs, this will be possible only as phenotypic information on more genes becomes available.

We believe that STIGMA is a valuable tool for clinical gene prioritization. Efforts like the Human Cell Atlas to map every cell type in the human body will further enhance STIGMA and other comparable tools.^82^

## Data and code availability

All the scripts used in this study for data preprocessing, parameter optimization, and building the random forest classifier are available for download at our GitHub repository https://github.com/SpielmannLab/STIGMA.

## Supplemental information

Document S1. Figures S1–S7

Table S1. List of genes used for training the model

Table S2. STIGMA-ranked genes for congenital limb malformations

Table S3. Monarch limb phenotypes for genes in STIGMA limb model

Table S4. STIGMA-ranked genes for congenital heart diseases

Document S2. Article plus supplemental information
