# STIGMA: Single-cell tissue-specific gene prioritization using machine learning

**DOI:** 10.1016/j.ajhg.2024.01.009

**Published:** 2024-03-07

**Authors:** Saranya Balachandran, Cesar A. Prada-Medina, Martin A. Mensah, Juliane Glaser, Naseebullah Kakar, Inga Nagel, Jelena Pozojevic, Enrique Audain, Marc-Phillip Hitz, Martin Kircher, Varun K.A. Sreenivasan, Malte Spielmann

## Main text

(The American Journal of Human Genetics *111*, 338–349; February 1, 2024)

As a result of an author oversight in the originally published version of this article, the author Juliane Glaser was missing. This error has now been corrected in the article online. The authors apologize for the error and any inconvenience that may have resulted.

