# Supplementary material for: STIGMA: Single-cell tissue-specific gene prioritization using machine learning
Source: Am J Hum Genet. 2024 Jan 15;111(2):338–49. doi: 10.1016/j.ajhg.2023.12.011 (PMC10870135; doi:10.1016/j.ajhg.2023.12.011)
Supplement: Document S1. Figures S1–S7 [file mmc1.pdf]

The American Journal of Human Genetics, Volume *111*

Supplemental information

STIGMA: Single-cell tissue-specific gene prioritization using machine learning

Saranya Balachandran, Cesar A. Prada-Medina, Martin A. Mensah, Juliane Glaser, Naseebullah Kakar, Inga Nagel, Jelena Pozojevic, Enrique Audain, Marc-Phillip Hitz, Martin Kircher, Varun K.A. Sreenivasan, and Malte Spielmann

Supplementary Information

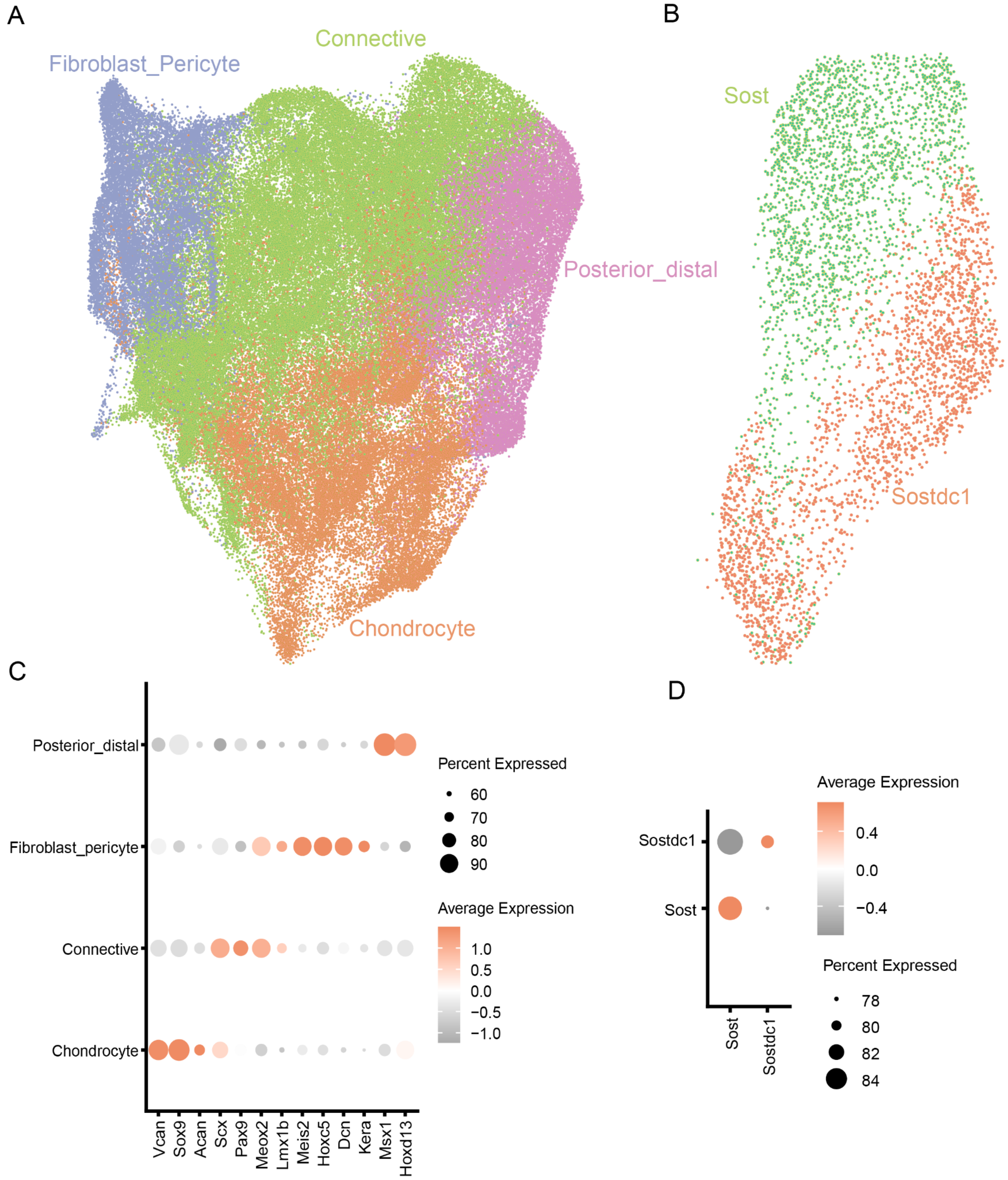

Figure S1. Sub-clustering of limb mesenchyme and ectoderm clusters. 2D UMAP embeddings of mesenchyme (**A**) and ectoderm (**B**) clusters coloured by sub-clusters, which were annotated based on the marker genes in **C**. and **D**. respectively.

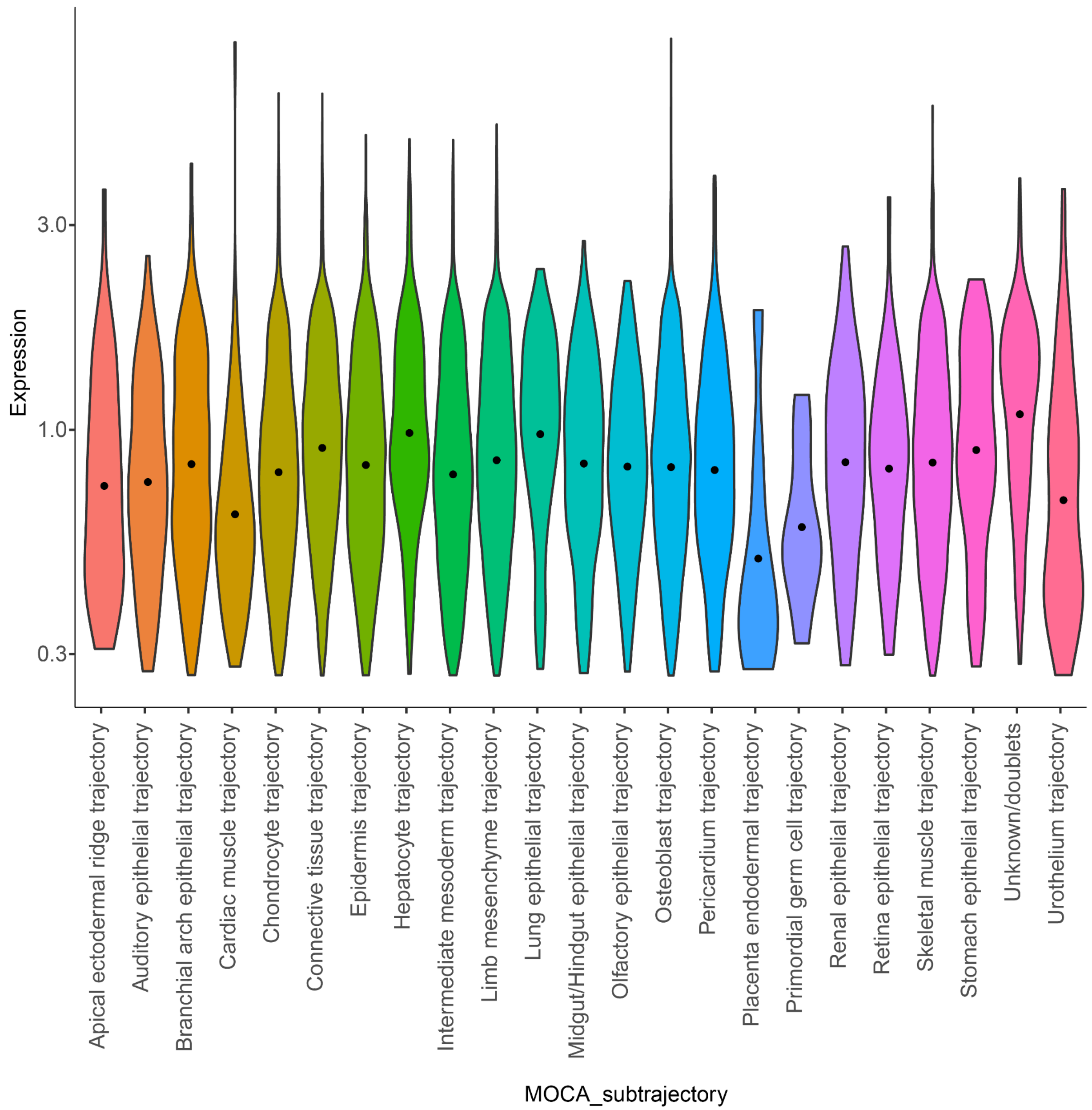

Figure S2. Some genes (e.g. *Hmgb1*) showed ubiquitous expression across all MOCA trajectories. The ubiquitous expression of *Hmgb1* across the sub trajectories of epithelial, hepatic, mesenchyme is shown as a representative example.

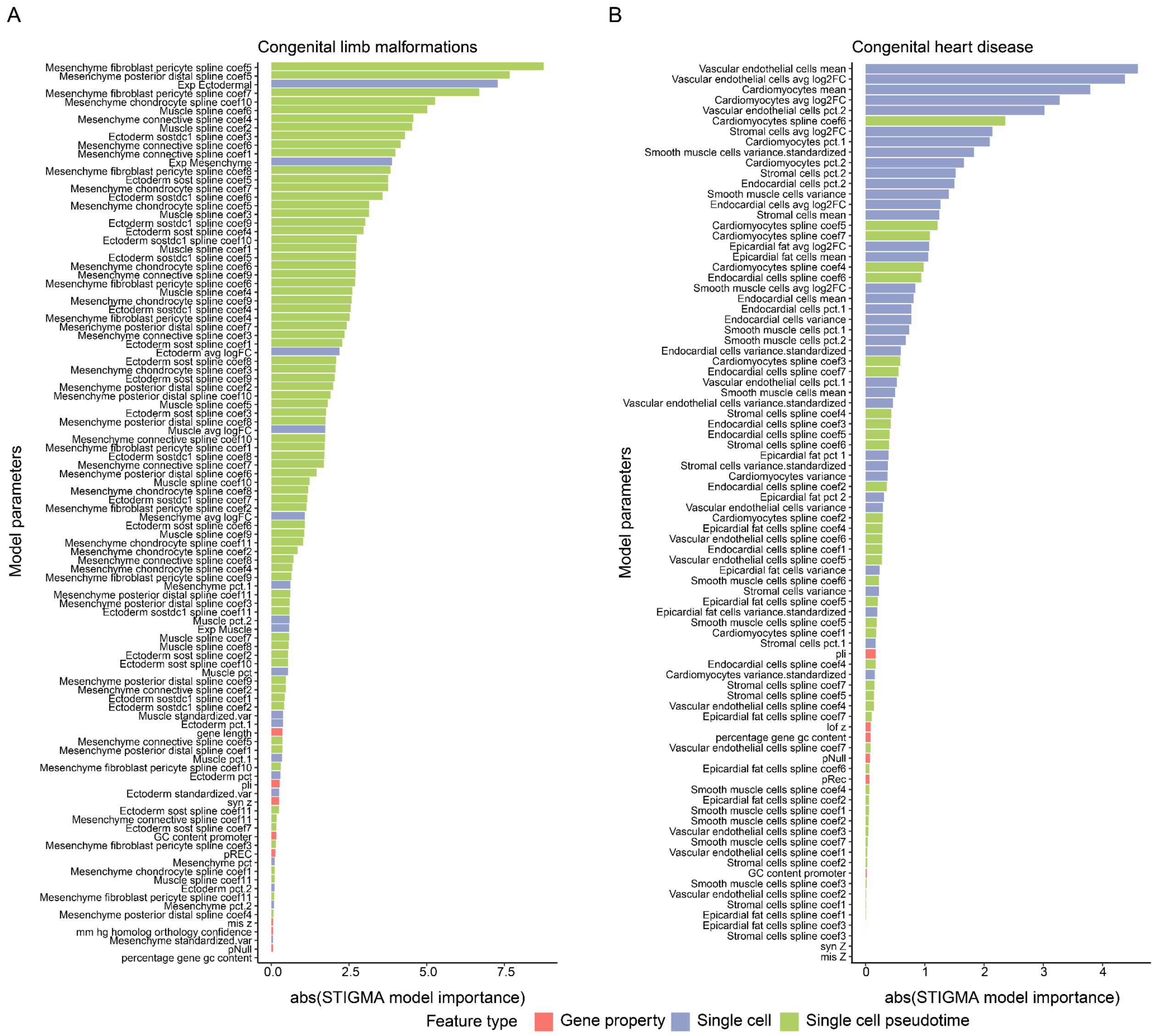

Figure S3. Importance of model features for STIGMA classification. The absolute values of the importance of model features for (**A**) congenital limb malformations and (**B**) congenital heart disease, where colors represent the feature type. The features labeled "Single cell" include cell type-specific features such as the percentage of cells expressing each gene and fold change in expression of a cell type compared to the rest of the cells, whereas "Single cell pseudotime" include spline coefficients of the temporal dynamics in expression per cell type.

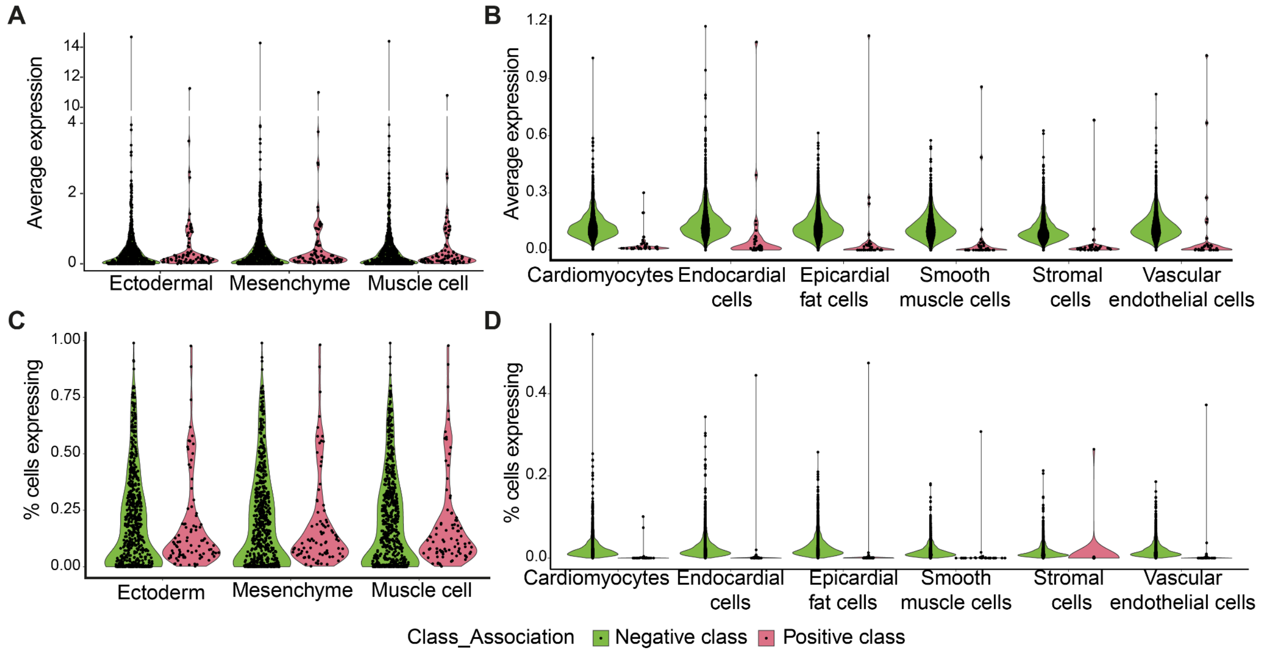

Figure S4. Expression of positive and negative class genes across the different cell types used in STIGMA. Average expression (**A**, **B**) and percentage of cells expressing (**C**, **D**) of the genes and in the limb (**A**, **C**) and heart (**B**, **D**) scRNA-seq datasets. The green and pink violins represent the distributions of the negative and positive class genes, respectively.

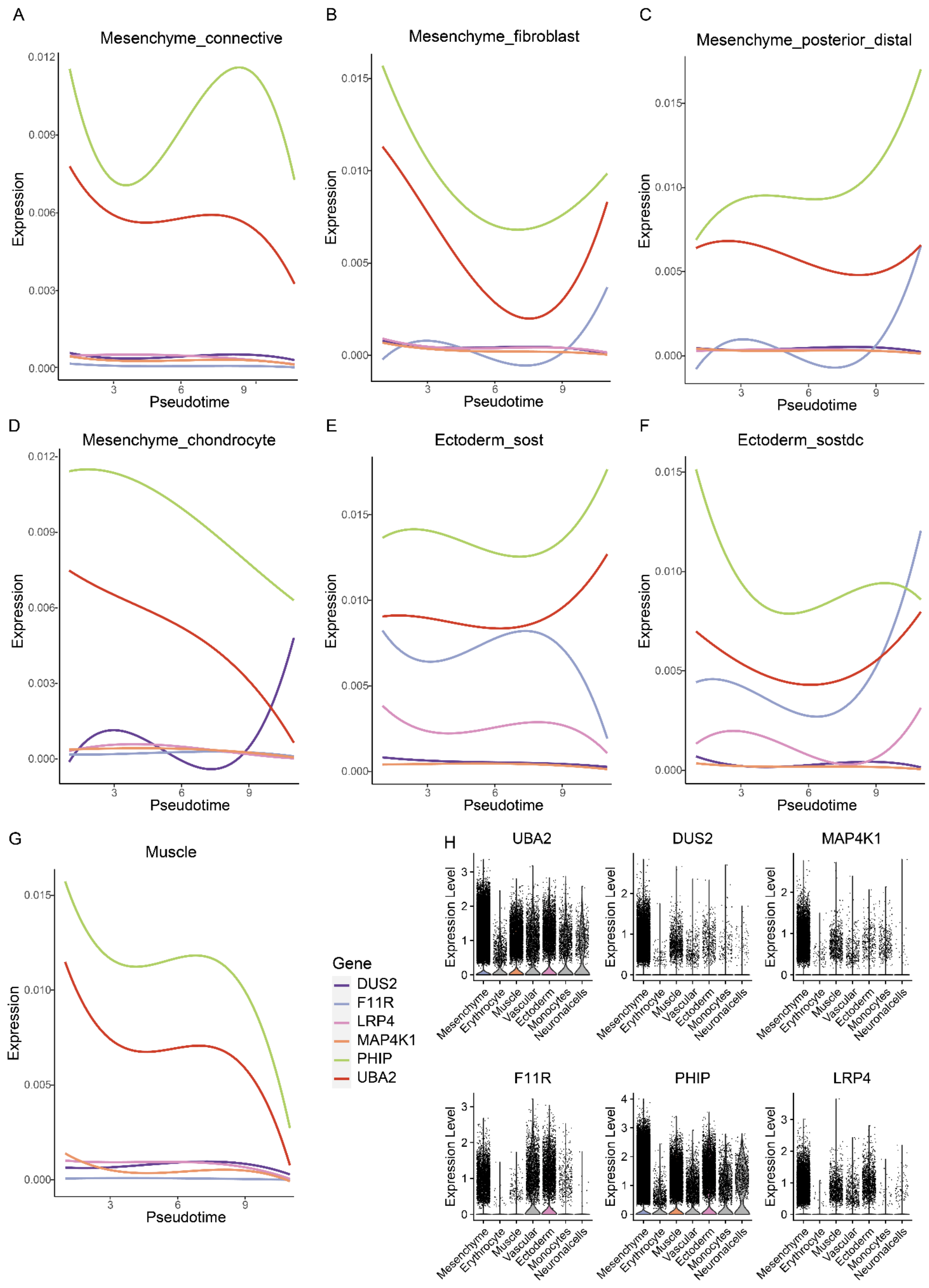

A
Mesenchyme_connective
B
Mesenchyme_fibroblast
C
Mesenchyme_posterior_distal
D
Mesenchyme_chondrocyte
E
Ectoderm_sost
F
Ectoderm_sostdc
G
Muscle
Expression
Pseudotime
Gene
DUS2
F11R
LRP4
MAP4K1
PHIP
UBA2
H
UBA2
DUS2
MAP4K1
F11R
PHIP
LRP4
Expression Level
Mesenchyme
Erythrocyte
Muscle
Vascular
Ectoderm
Monocytes
Neuronalcells

Figure S5. Temporal dynamics and cellular expression of genes with *de novo* mutations. A-G. Dynamics in the expression of genes with potential LoF identified in the cohort of congenital limb malformations along the developmental pseudo-time in limb sub-trajectories [30]. The lines represent spline fits. **H**. Average expression of the genes across cellular clusters. Cell types not used for training STIGMA are grayed out.

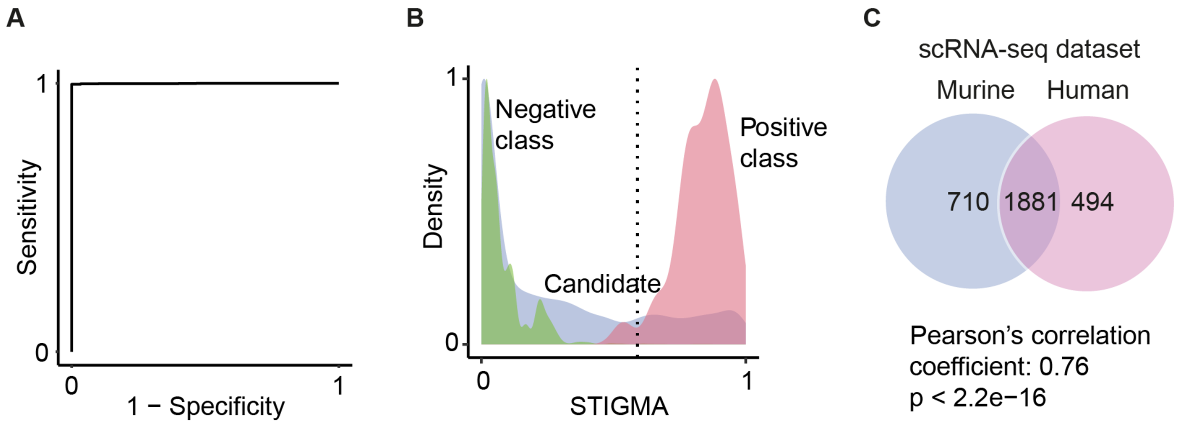

Figure S6: Performance of STIGMA for congenital heart disease trained on murine dataset and its comparison to that trained with human dataset. A. ROC curve showing the performance of the model. **B.** Distribution of STIGMA scores for training class and candidate genes. Dotted line marks the binary classification threshold of 0.59. **C.** Venn Diagram showing the number of genes predicted by using the murine and human scRNA-seq fetal heart datasets. Pearson's correlation coefficient, R, between the STIGMA scores of genes by training with murine and human datasets and the corresponding p-value are shown. scRNA-seq data for the murine dataset (E14.5 - E18) were downloaded from Feng et al [83] and processed as described in Methods. Note, 4622 genes in the human dataset were not present in the murine dataset, of which some are due to the absence of orthologs.

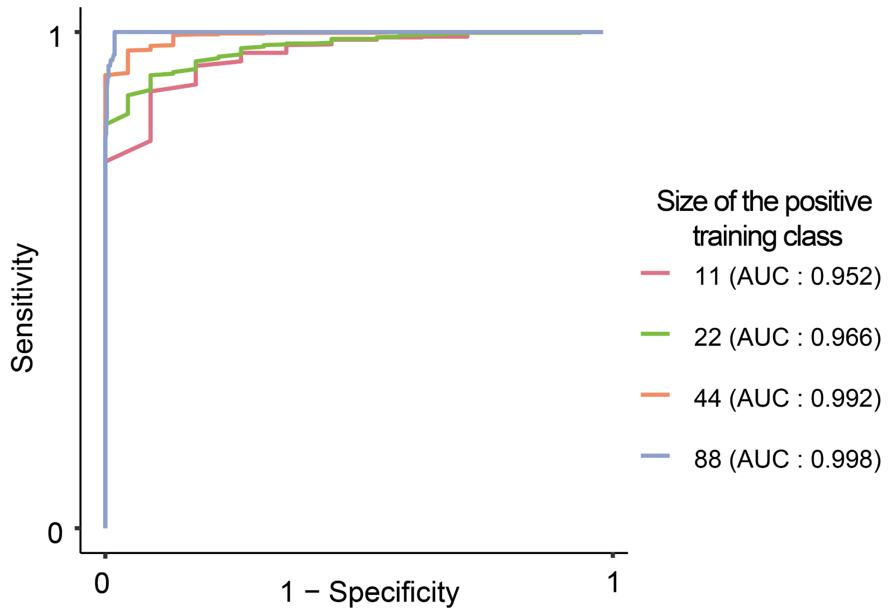

Figure S7. Stress test of STIGMA performance as the size of the positive training class reduces. The ROC curves of STIGMA binary classifiers trained with downsampled positive classes. The corresponding AUC are shown in the legends.

Supplementary Tables

Table S1. List of genes used for training the model

Table S2. STIGMA-ranked genes for congenital limb malformations

Table S3. Monarch limb phenotypes for genes in STIGMA limb model

Table S4. STIGMA-ranked genes for congenital heart diseases
